# Predicting soil chemical characteristics in the arid region of central Iran using remote sensing and machine learning models

**DOI:** 10.1038/s41598-025-04554-8

**Published:** 2025-07-02

**Authors:** Azita Molaeinasab, Hossein Bashari, Mostafa Tarkesh Esfahani, Saeid Pourmanafi, Norair Toomanian, Bahareh Aghasi, Ahmad Jalalian

**Affiliations:** 1 Department of Natural Resources, Isfahan University of Technology, Isfahan, 84156-83111 Iran; 2 Soil and Water Research Department, Isfahan Agricultural and Natural Resources Research and Education Center, AREEO, Isfahan, Iran; 3 Department of Natural Resources and Watershed Management of Isfahan Province, Isfahan, 8174679581 Iran; 4 Department of Soil Science, Islamic Azad University, Isfahan (Khorasgan) Branch, Isfahan, 8155139998 Iran

**Keywords:** Digital soil mapping, Ensemble modeling, Spectral indices, Variable selection (Boruta), Environmental covariates, Cross-validation, Ecological modelling, Solid Earth sciences, Wetlands ecology

## Abstract

Digital Soil Mapping (DSM) techniques have advanced significantly in recent decades, helping to close critical gaps in soil data and knowledge. This study was conducted in the arid Gavkhouni sub-basin of Isfahan Province, central Iran, where environmental stresses such as salinity and water scarcity challenge sustainable land management. We employed 34 environmental covariates derived from Landsat 8 imagery and a digital elevation model, combined with 96 surface soil samples (0 to 20 cm depth), to assess the performance of six machine-learning models: Random Forest (RF), Classification and Regression Tree (CART), Support Vector Regression (SVR), Generalized Additive Model (GAM), Generalized Linear Model (GLM), and an ensemble approach. Unlike many previous studies that have focused on a single soil attribute with a limited set of predictors, our work adopts an integrated approach to map four salinity-related soil properties: Ca, CaCO_3_, CaSO_4_, and SO_4_. Predictor selection involved multicollinearity testing using the Variance Inflation Factor (VIF) and the Boruta algorithm. Model performance was assessed using tenfold cross-validation. The ensemble model performed best, achieving R^2^ values of 0.89 for Ca, 0.84 for CaCO_3_, 0.79 for SO_4_, and 0.73 for CaSO_4_. Elevation and the Temperature-Vegetation Dryness Index (TVDI) were the most influential predictors for Ca, while the Tasseled Cap Brightness (TCB) and Tasseled Cap Wetness (TCW) indices were most important for CaCO_3_. For CaSO_4_, Band 5 (B5) and TCB were the most effective, whereas SO_4_ predictions were driven by TCB along with Bands 5 and 7. These findings highlight the potential of remote sensing-based DSM to enhance soil monitoring in data-scarce, arid environments. The growing availability of free satellite data, such as Landsat, offers valuable opportunities to improve soil assessment and promote sustainable land management in resource-limited regions like Iran.

## Introduction

High-resolution soil mapping is essential for effective environmental management as it provides critical insights into soil characteristics and supports strategies to address ecological challenges. By identifying regions at risk of degradation, such mapping supports the conservation of soil biodiversity and informs sustainable land-use practices, contributing to long-term ecosystem health^1,2^.

Traditionally, soil maps have been generated through ground surveys and other conventional techniques^3^. While valuable, such methods often lack the spatial resolution and efficiency needed to capture fine-scale variability, especially across large and environmentally diverse landscapes. Limitations in field coverage, high implementation costs, and reduced practicality in remote or arid regions make them less suitable for large-scale applications^4–8^.

Digital Soil Mapping (DSM) has emerged as a more scalable and accurate alternative by integrating remote sensing (RS) data and machine learning (ML) techniques. Rooted in the SCORPAN framework, an extension of Jenny’s^9^ CLORPT model, DSM leverages environmental covariates such as soil properties, climate, organisms, topography, parent material, time, and spatial context to estimate soil characteristics over broad regions^5^. This approach facilitates efficient prediction of soil attributes at finer resolutions, addressing many of the limitations of traditional methods.

Topographic features and multispectral indices derived from satellite imagery, such as those from Landsat 8, MODIS, and Sentinel-2, are particularly influential predictors in DSM^10–12^. Remote sensing data provides a reliable, cost-effective means to analyze spectral reflectance patterns and landscape features across extensive and often inaccessible terrains. By offering consistent and repeatable measurements over time, RS reduces reliance on intensive field campaigns and enhances the spatial and temporal coverage of soil information^6,13–16^.

Building on these global advances, several studies in Iran have employed remote sensing and ML approaches to model key soil properties. For example, Faramarzi et al.^17^ used Random Forest (RF) and artificial neural networks (ANN) to estimate soil organic matter content (SOMC) in irrigated lands, demonstrating superior performance of RF when combining spectral and topographic predictors. Similarly, Khosravi Aqdam et al.^18^ assessed soil fertility across elevation gradients in northwestern Iran, revealing significant altitudinal effects on macronutrient and micronutrient distributions. These findings highlight the value of environmental covariates, especially terrain metrics, in explaining soil variability and guiding soil monitoring efforts.

Although many recent studies have adopted ML-assisted DSM to map various soil properties^19^, Amindin et al.^20,21^, they often focus on single soil attributes or utilize a narrow set of predictors. To address these limitations, this study applies a broader modeling framework to explore complex soil-environment interactions using multiple algorithms.

We employed a diverse set of machine learning models including Generalized Linear Models (GLM), Generalized Additive Models (GAM), Classification and Regression Trees (CART), Random Forest (RF), Support Vector Regression (SVR), and an ensemble learning approach to evaluate their relative performance in DSM. GLM and GAM offer parametric and semi-parametric perspectives for modeling linear and non-linear trends^22,23^. CART and RF, as decision-tree-based models, excel in handling complex interactions and noisy datasets^24^. SVR provides a robust kernel-based technique well-suited for high-dimensional feature spaces^25^. The ensemble model integrates the outputs of these diverse algorithms to capitalize on their complementary strengths and improve overall predictive performance.

A significant portion of Iran, particularly its central plateau, is increasingly affected by environmental stressors such as soil and water salinity, drought, desertification, and land degradation. The Gavkhouni wetland, a unique ecosystem characterized by an arid climate, is among the most ecologically sensitive areas in the region^26^. Declining water inflow, overgrazing, pollution, and land-use changes have intensified the need for accurate soil assessment and sustainable land management^27^, Pirali Zefrehei et al.^28^.

Among the critical soil properties influencing land degradation and salinization in arid regions are calcium (Ca), calcium carbonate (CaCO_3_), calcium sulfate (CaSO_4_), and sulfate (SO_4_). These chemical components are key indicators of soil salinity and sodicity levels. For example, SO_4_ is a major anion in many saline soils, while Ca^2^⁺ plays a central role in flocculating clay particles and counteracting sodium-induced dispersion. CaCO_3_ affects soil buffering capacity and nutrient availability, and CaSO_4_ (gypsum) is widely used to reclaim sodic soils. Understanding the spatial distribution of these elements provides essential information for managing saline soils, improving land productivity, and preventing further soil degradation in vulnerable ecosystems^29^.

Unlike previous studies that typically focus on a single soil property or a narrow set of predictors, this research simultaneously maps four salinity-related chemical indicators (Ca, CaCO_3_, CaSO_4_, and SO_4_) using a rich dataset of 34 environmental covariates derived from digital elevation models and remote sensing indices. The core research question explores the extent to which these properties can be accurately predicted using a diverse suite of machine learning algorithms in an arid region of central Iran.

The novelty of this study lies in its integrated approach, which combines multiple chemical parameters, advanced ensemble learning techniques, and a wide range of environmental predictors to enhance spatial detail and prediction accuracy. This method offers deeper insight into the spatial dynamics of soil salinity and provides practical tools for soil conservation and land-use planning in vulnerable ecosystems.

The main objectives of this study include: (a) spatially predicting four soil properties using five statistical models, and an ensemble model, (b) evaluating the performance of these machine learning models in soil property prediction, (c) identifying the most important environmental auxiliary variables influencing each soil property, and (d) producing detailed soil property maps based on the best-performing machine learning model.

## Materials and methods

### Study area and data collection

**Fig. 1:**
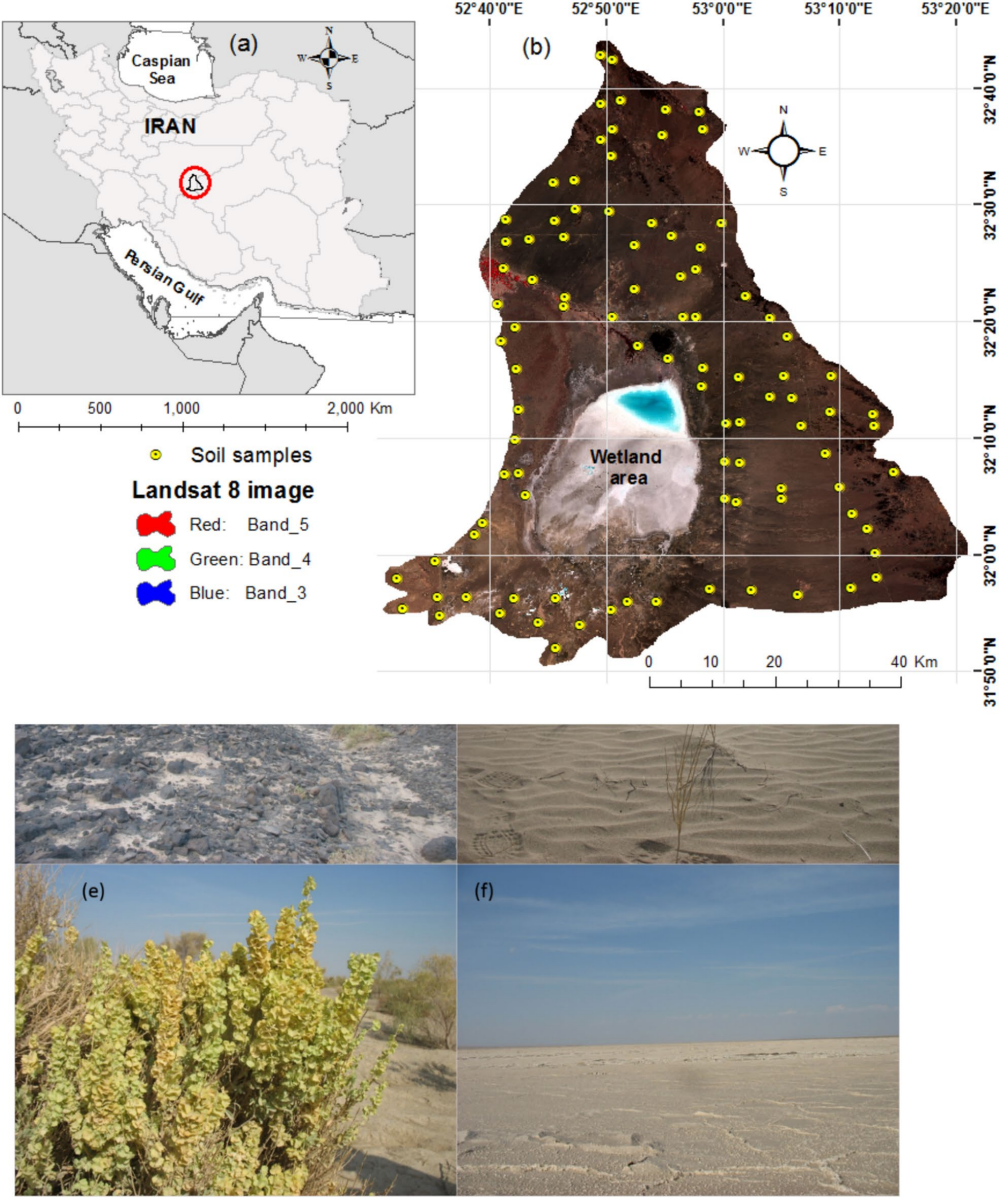
(**a**) Location of the Gavkhouni sub-basin, Isfahan, Iran; (**b**) Study area shown in a Landsat 8 false-color composite image (autumn 2013) with RGB representation of Near Infrared, Red, and Green, alongside the distribution of soil samples; (**c**) Koh-e-Siah (Black Mountain), a volcanic formation with andesite outcrops; (**d**) Sand dunes with scattered vegetation; (**e**) Restored site with planted vegetation; (**f**) Salt pans in the Gavkhouni International Wetland.

This study was conducted in the desert rangelands surrounding the Gavkhouni Wetland, encompassing an area of 3808 square kilometers. The region is located southeast of Isfahan, in the eastern portion of the Zayandeh Rud watershed within Iran’s central plateau (Fig. 1). The Gavkhouni Wetland sub-basin comprises a single hydrological unit known as the Gavkhouni Hydrological Unit, with most of the sub-basin characterized by plains. The highest point in the sub-basin, located in the northern part of the region, reaches an elevation of 2461 m above sea level, while the lowest point is at the Gavkhouni Wetland, at approximately 1450 m. The average elevation of the sub-basin is calculated at 1549 m.

The region is characterized by an arid desert climate, with an average annual precipitation of 83 mm, an average temperature of 19 °C, and an annual potential evapotranspiration of 3265 mm. Based on the De Martonne classification, the climate is categorized as arid desert. The area’s temperature classification falls under the thermic regime, while the soil moisture regime is classified as arid, with Aridisols being the dominant soil order^30,31^.

South of the study area lies the internationally recognized Gavkhouni Wetland, covering 472 square kilometers, which is considered morphologically part of Iran’s central playas. The region’s dominant vegetation includes species such as *Haloxylon ammodendron* (C.A.Mey.) Bunge ex Fenzl., *Tamarix* spp., *Seidlitzia rosmarinus* Bunge ex Boiss., *Halocnemum strobilaceum* (Pall.) M.Bieb., and *Halostachys caspica* C.A. Mey. Sand dunes are also present to the west of the wetland (Fig. 1).

**Fig. 2:**
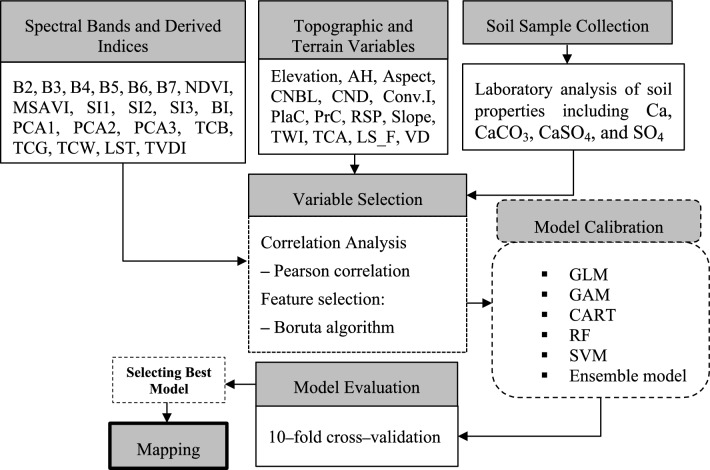
Workflow of digital soil mapping using remote sensing, topographic data, and machine learning models.

A schematic overview of the methodological workflow is presented in Fig. 2. This flowchart visually summarizes the key steps undertaken in the study, including data collection, environmental variable preparation, model development, and validation. Each phase is further detailed in the subsequent sections to provide a comprehensive understanding of the procedures applied.

To ensure representative coverage of the study area’s considerable size (3616 km^2^), a hybrid approach combining grid-based sampling and proportional stratified random sampling was adopted. The Gavkhouni sub-basin was partitioned into 100 grid cells, each covering 36 km^2^ (6 × 6 km). Sampling strata were defined based on distinct geomorphological units identified within the region. The number of samples allocated to each stratum was proportional to its area, ensuring that the spatial distribution of samples reflected the varying sizes of these landform units. Within each geomorphological unit, random sampling was used to determine precise locations, promoting unbiased data collection across different terrains. In total, 96 surface soil samples were collected from the top 0–20 cm layer. This sampling depth was selected to align with the topsoil zone commonly assessed in soil quality and mapping studies. Each sampling point was georeferenced using GPS. Laboratory analysis included the quantification of Ca, CaCO_3_, CaSO_4_, and SO_4_, following the protocols outlined by Carter and Gregorich^32^. The soil sampling campaign was synchronized with satellite data acquisition to facilitate integrated analysis.

### Environmental variables

In this study, remote sensing data and topographic features were utilized as auxiliary variables to develop models for generating digital maps of four soil properties. The primary source of remote sensing data comprised cloud-free images captured by the OLI/TIRS sensor of the Landsat 8 satellite during autumn 2013. To derive soil parameter maps, remote sensing data including various bands, Tasseled Cap Transformation (TCT)^33^, salinity indices^34^, and principal component analysis (PCA) were used^35^. The selected remote sensing environmental variables included three visible bands (bands 2, 3, and 4), near-infrared (band 5), shortwave infrared (bands 6 and 7), and longwave infrared (band 10), all with a spatial resolution of 30 m. The bands and indices were employed to capture a range of soil characteristics, with the Tasseled Cap coefficients for brightness, greenness, and wetness calculated using the formulas provided by Baig et al.^33^. Additionally, several salinity indices were derived: Soil Salinity Index 1 (SI1) and Soil Salinity Index 2 (SI2) from Khan et al.^36^ and Douaoui and Lepinard^37^, respectively, and Soil Salinity Index 3 (SI3) as per Douaoui et al.^38^. The Normalized Difference Vegetation Index (NDVI) and Modified Soil-Adjusted Vegetation Index (MSAVI) were computed using the approaches detailed by Rouse et al.^39^ and Qi et al.^40^, respectively. The Land Surface Temperature (LST) and the Temperature Vegetation Drought Index (TVDI) were derived from Landsat 8 imagery using the calculations outlined by Ermida et al.^41^ and Han et al.^42^ (refer to Appendix 1 for the formula of these remote sensing indices). These indices and variables were integrated into the soil property models to enhance their accuracy and relevance.

Another important environmental variable was the Digital Elevation Model (DEM), sourced from the Shuttle Radar Topography Mission (SRTM) at a 30-m resolution. The DEM was refined using the ‘filling sinks’ function in SAGA GIS software, based on the method by Planchon and Darboux^43^. As a 3D representation of Earth’s surface, the DEM offers crucial altitude data relative to sea level, serving as a valuable resource for assessing surface changes and understanding geological processes.

Topographic features for the soil characteristic models were derived from various environmental variables based on the DEM, such as elevation, slope, aspect, topographic wetness index (TWI), length-slope factor (LS_F), analytical hillshading (AH), channel network base level (CNBL), channel network distance (CND), convergence index (Conv.I), plan curvature (PlaC), profile curvature (PrC), relative slope position (RSP), total catchment area (TCA), and valley depth (VD) (Appendix 2). These topographic variables were generated using SAGA GIS software^44^, with computational details provided by Hengl et al.^45^. Additionally, relief factors derived from the 30-m resolution SRTM DEM included thirteen elevation derivatives calculated through SAGA GIS (Appendix 2).

### Variable selection method

Given the large number of candidate auxiliary variables (34 in total), a systematic variable selection procedure was adopted to ensure model parsimony and minimize multicollinearity. Initially, Pearson correlation analysis was conducted to assess the strength and direction of associations between environmental variables and each soil parameter targeted for modeling.

Subsequently, two complementary approaches were applied within the R statistical environment: the Variance Inflation Factor (VIF) method, using the ‘usdm’ package^46,47^, and the Boruta feature selection algorithm, implemented through the ‘Boruta’ package^48^.

The VIF method was employed as a stepwise elimination procedure to address multicollinearity by iteratively removing variables exhibiting high redundancy. Following standard practice, auxiliary variables with VIF values ≥ 10 were systematically excluded, a threshold commonly recommended to identify problematic multicollinearity^46,49^. This process resulted in the retention of 22 environmental predictors with acceptable levels of collinearity.

To further refine the predictor set and enhance model robustness, the Boruta algorithm was applied. Boruta operates by iteratively comparing the importance of original variables against randomly permuted ‘shadow’ variables to determine genuine predictive relevance. Using the default iteration settings, Boruta classified each variable as important, unimportant, or tentative based on its contribution to the response variables. This two-step variable selection approach minimized bias, reduced model complexity, and improved the reliability of subsequent analyses.

### Importance of predictor variables

In this study, the importance of predictor variables was evaluated using the "varImp()" function from the *caret* package in R, which ranks variables based on their contribution to the model’s predictive accuracy. After the model was fitted, "varImp()" computed the importance scores by leveraging internal mechanisms of the algorithm, such as reductions in accuracy or impurity in decision tree models, or the magnitude of coefficients in linear models. This approach provided a clear view of the influence each variable had on the prediction results, highlighting the most significant environmental and topographic factors. The selected variables with the highest importance scores were subsequently utilized to improve the precision of predicting key soil properties, such as CaCO_3_, CaSO_4_, SO_4_, and Ca, by prioritizing those that contributed most to the model’s performance.

### Modeling approaches

In this research, five different machine learning models were applied: Generalized Linear Model (GLM), Generalized Additive Model (GAM), Classification and Regression Trees (CART), Random Forest (RF), Support Vector Regression (SVR), along with an Ensemble model. These models were chosen to investigate the links between soil properties and auxiliary environmental variables, with implementation carried out using the “Caret” package in R^50^. A brief explanation of each model is provided below.

GLM extends traditional linear regression by supporting response variables that may follow different types of distributions. It connects the expected value of the dependent variable with a linear function of the predictor variables. Through the use of link functions, GLM enables the model to manage various data types by transforming predictions^51^. In this study, the Gaussian link function was applied to model continuous variables^22,52^.

GAM builds on the GLM framework but introduces flexibility by incorporating smooth, non-linear functions of predictor variables. It is particularly suitable when relationships between predictors and the response are complex and non-linear. Unlike GLM, which assumes linearity, GAM provides a semi-parametric approach, fitting smoother functions to capture intricate patterns in the data^23^.

CART is a decision-tree-based algorithm that splits data into branches to predict outcomes. It operates through recursive partitioning, creating a tree structure where each node represents a split based on a particular variable. This method is valued for its interpretability and simplicity, allowing straightforward representation of both regression and classification tasks^53^. For implementation in R, the “rpart” package was used to fit the model^54^.

RF enhances the predictive power of decision trees by generating a multitude of trees and averaging their results. This ensemble approach reduces overfitting and improves model accuracy by bootstrapping the data to grow multiple trees. Each tree contributes a prediction, with the final output being either the majority vote (classification) or the average prediction (regression)^24^. RF is particularly effective for handling high-dimensional and noisy data, making it well-suited for this study’s complex environmental predictors^55^. The model was optimized through key parameters such as the number of trees and the number of variables considered at each split^56^. For this study, the RF model used 500 trees and variable selection based on multiple tuning parameters.

SVR is a versatile machine learning algorithm that constructs hyperplanes to separate data points into distinct classes or fit data for regression tasks. In regression analysis, SVR seeks to minimize prediction errors within a specified tolerance. The algorithm performs exceptionally well in capturing complex relationships in high-dimensional data^57^. For this research, an RBF kernel was applied, tuning parameters such as the cost (C) and gamma^58^, which control the complexity and regularization of the model.

The ensemble model synthesizes predictions from several individual models to enhance overall performance in predicting soil chemical properties. By combining the results of RF, CART, and SVR, each weighted according to its predictive strength, this approach captures diverse patterns within the data and reduces prediction error. To ensure robustness, models with R-squared values exceeding 0.5 were selected for inclusion in the ensemble. The weights for each model were determined based on their R^2^ values, which were calculated as a measure of the model’s ability to explain variance in the observed data. This method ensures that models with higher R^2^ values contribute more substantially to the final ensemble prediction. The ensemble prediction for each soil property was computed using a weighted averaging formula (Eq. 1), commonly used in ensemble modeling frameworks (e.g^59,60^.,):

1 $${\text{S}}_{\text{ensemble}}=\frac{{\sum }_{i=1}^{n}{w}_{i}\times {S}_{i}}{{\sum }_{i=1}^{n}{w}_{i}}$$

Here, S_ensemble_ represents the aggregated prediction for the soil property, S_i_ denotes the prediction from model *i*, and w_i_ is the weight assigned to model *i* based on its R^2^ value.

### Model validation

The accuracy and predictive capability of the machine learning models were evaluated by applying three statistical indicators: Root Mean Square Error (RMSE), Mean Absolute Error (MAE), and the coefficient of determination (R^2^). These metrics quantify how well the models predicted soil properties and are standard in model validation^61^. To ensure strong validation, we applied tenfold cross-validation using the “caret” package in R. The dataset was randomly split into ten equal portions. In each iteration, one portion was reserved for testing while the other nine were used for training. This procedure was repeated ten times, ensuring each portion was used as a test set once. The average performance across all folds is then calculated, providing an unbiased estimate of the model’s generalization ability. This method is especially beneficial for smaller datasets as it maximizes the use of available data without overfitting. The accuracy of the models was measured by calculating the RMSE, MAE, and R^2^ for each fold. RMSE measures how well the model minimizes larger prediction errors, while MAE offers the average magnitude of all errors without considering their direction. R^2^ indicates the proportion of variation in the observed data that is accounted for by the model. The following equations represent the statistical parameters used to evaluate the models:

2 $${\text{R}}^{2}=1-\frac{\sum {({y}_{i}-\widehat{y})}^{2}}{\sum {({y}_{i}-\overline{y })}^{2}}$$

3 $$R\text{MSE}=\sqrt{\frac{1}{n}\sum_{i=1}^{n}{({y}_{i}-\widehat{y})}^{2}}$$

4 $$\text{MAE}=\frac{1}{\text{n}}{\sum }_{\text{i}=1}^{\text{n}}\left|{\text{y}}_{\text{i}}-\widehat{y}\right|$$

where $\widehat{y}$ represents the predicted values, ${\text{y}}_{\text{i}}$ is the observed values, and $\overline{y }$ is the mean of the observed values. These metrics provide a thorough assessment of the model’s predictive accuracy, enabling a clear understanding of its strengths and limitations.

## Results

### Descriptive statistics soil data

Descriptive statistics for the four measured soil chemical properties including calcium (Ca), calcium carbonate (CaCO_3_), calcium sulfate (CaSO_4_), and sulfate (SO_4_) are presented in Table 1. The data show substantial variability across all parameters. Calcium concentrations ranged from 1.00 to 185.00 meq/L, with a mean of 25.68 meq/L and a high coefficient of variation (CV = 140.22 percent). The distribution exhibited strong positive skewness (2.43) and elevated kurtosis (6.25), indicating a right-skewed, leptokurtic pattern. Calcium carbonate values ranged from 2.97 percent to 51.36 percent, with a mean of 26.04 percent and moderate variability (CV = 38.10 percent). The distribution was approximately symmetric, with low skewness (0.63) and kurtosis (0.21), suggesting near-normal behavior. In contrast, calcium sulfate showed extreme variability, with values ranging from 0.00 percent to 50.42 percent. The mean was 1.72 percent, the standard deviation was 5.44 percent, and the coefficient of variation was 316.31 percent. The distribution was highly skewed (skewness = 7.88) and sharply peaked (kurtosis = 69.27). Sulfate concentrations varied from 0.99 to 452.04 meq/L, with a mean of 45.42 meq/L and a high coefficient of variation (151.72 percent). The distribution was positively skewed (3.50) and leptokurtic (14.83). According to the classification proposed by Wilding^62^, all variables exhibited high spatial variability, as indicated by CV values greater than 35 percent. Frequency histograms (Appendix 3) confirm the non-normal, right-skewed distributions of Ca, CaSO_4_, and SO_4_. These patterns reflect the pronounced heterogeneity and extreme variability of these soil chemical properties across the study area.

**Table 1:** Summary statistics of observed soil properties for total data set (N = 96).

Soil parameters	Unit	Min	Max	Mean	StDev	CoefVar (%)	Skew	Kurt	Q25	Q50	Q75
Ca	meq/L	1.00	185.00	25.68	36.01	140.22	2.43	6.25	3.20	13.30	32.00
CaCO_3_	%	2.97	51.36	26.04	9.92	38.10	0.63	0.21	20.27	23.74	29.76
CaSO_4_	%	0.00	50.42	1.72	5.44	316.31	7.88	69.27	0.28	0.60	1.10
SO_4_	meq/L	0.99	452.04	45.42	68.92	151.72	3.50	14.83	6.16	31.14	44.52

Min, minimum; Max, maximum; StDev, standard deviation; CoefVar, coefficient of variation; Skew, Skewness; Kurt, Kurtosis; Q25 = 25th percentile; Q50 = median; Q75 = 75th percentile.

**Fig. 3:**
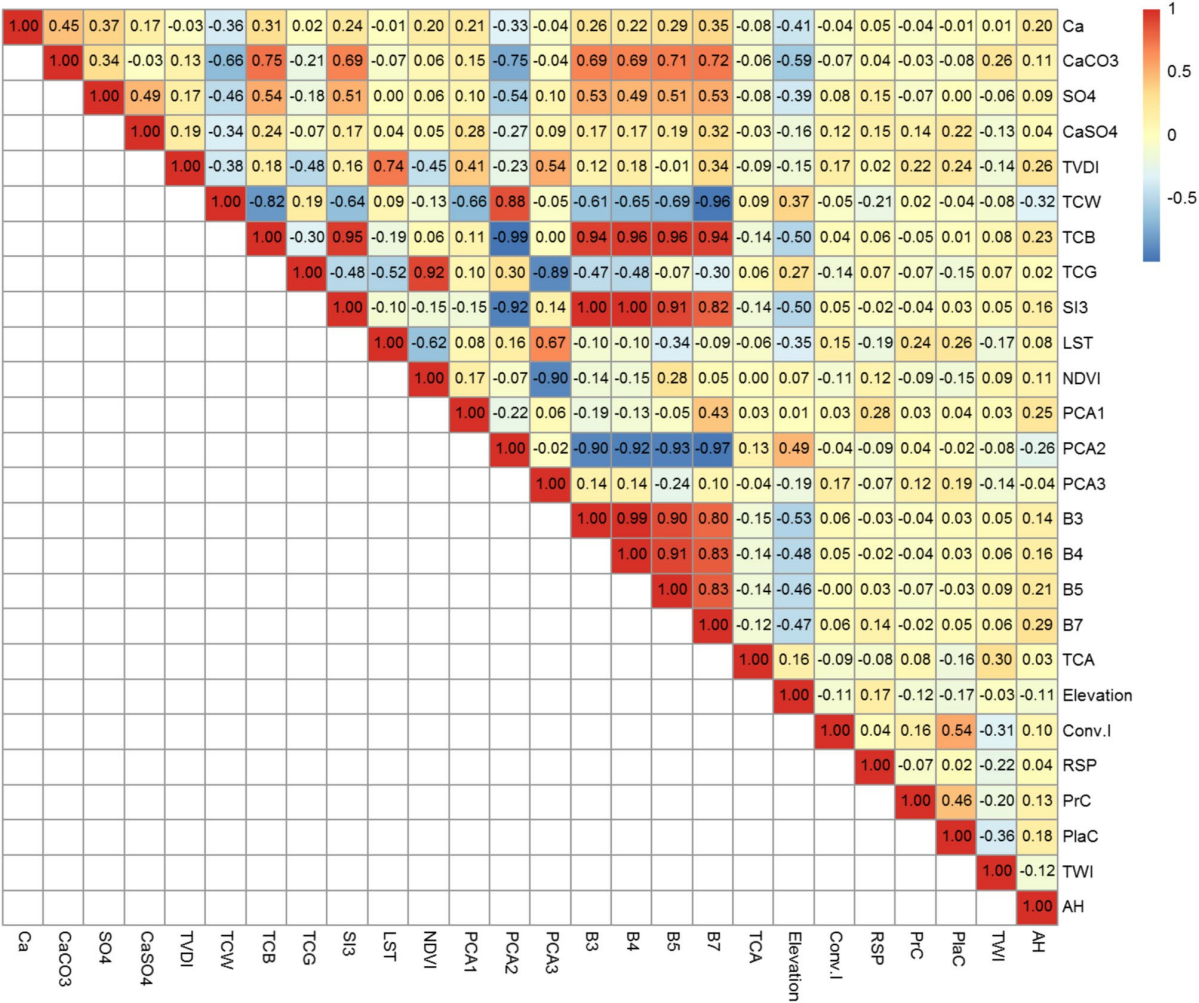
Two-dimensional heatmap based on the correlation matrix of studied variables. Weak correlations are displayed in low color intensity, while stronger ones are shown with high color intensity. Positive correlations were represented by red color, while negative ones were marked in blue.

To evaluate how sensitive Landsat OLI-derived variables (such as spectral bands, PCA images, vegetation indices, tasseled cap transformation, and satellite-based salinity indices) are to different soil properties, Pearson correlation analysis was utilized. This analysis helped to identify the most effective predictors for the modeling process. As shown in Fig. 3, significant correlations were found between the 22 covariates derived from Landsat and various soil properties. Many of these variables demonstrated strong correlations with the soil components Ca, CaCO_3_, CaSO_4_, and SO_4_ in the study area.

The Pearson correlation matrix (Fig. 3) reveals notable correlations among the predictor variables, with the most prominent relationships occurring between bands 2–7 and the salinity indices (SI1, SI2, and SI3). Despite these strong correlations, these predictors are not considered redundant, as they capture different yet complementary aspects of soil characteristics. Other correlations among predictor variables were moderate to low.

The weakest correlations were found among certain soil properties. However, CaCO_3_ showed a strong positive association with indices TCB, B7, B5, B3, B4, and SI3. In contrast, a strong negative association was observed between CaCO_3_ and indices PCA2 and TCW. Similarly, SO_4_ showed significant positive correlations with indices B7, TCB, SI3, B3, and B5, while a negative correlation was noted between SO_4_ and PCA2.

### Covariate data selection and importance

Variable selection was conducted using the VIF test and the Boruta algorithm, resulting in the retention of 22 environmental variables for the modeling process. Specifically, out of the initial 34 variables, 8 topographic and 14 remote sensing variables were identified as the most relevant predictors. The details of the selected variables are provided in Appendices 1 and 2.

**Fig. 4:**
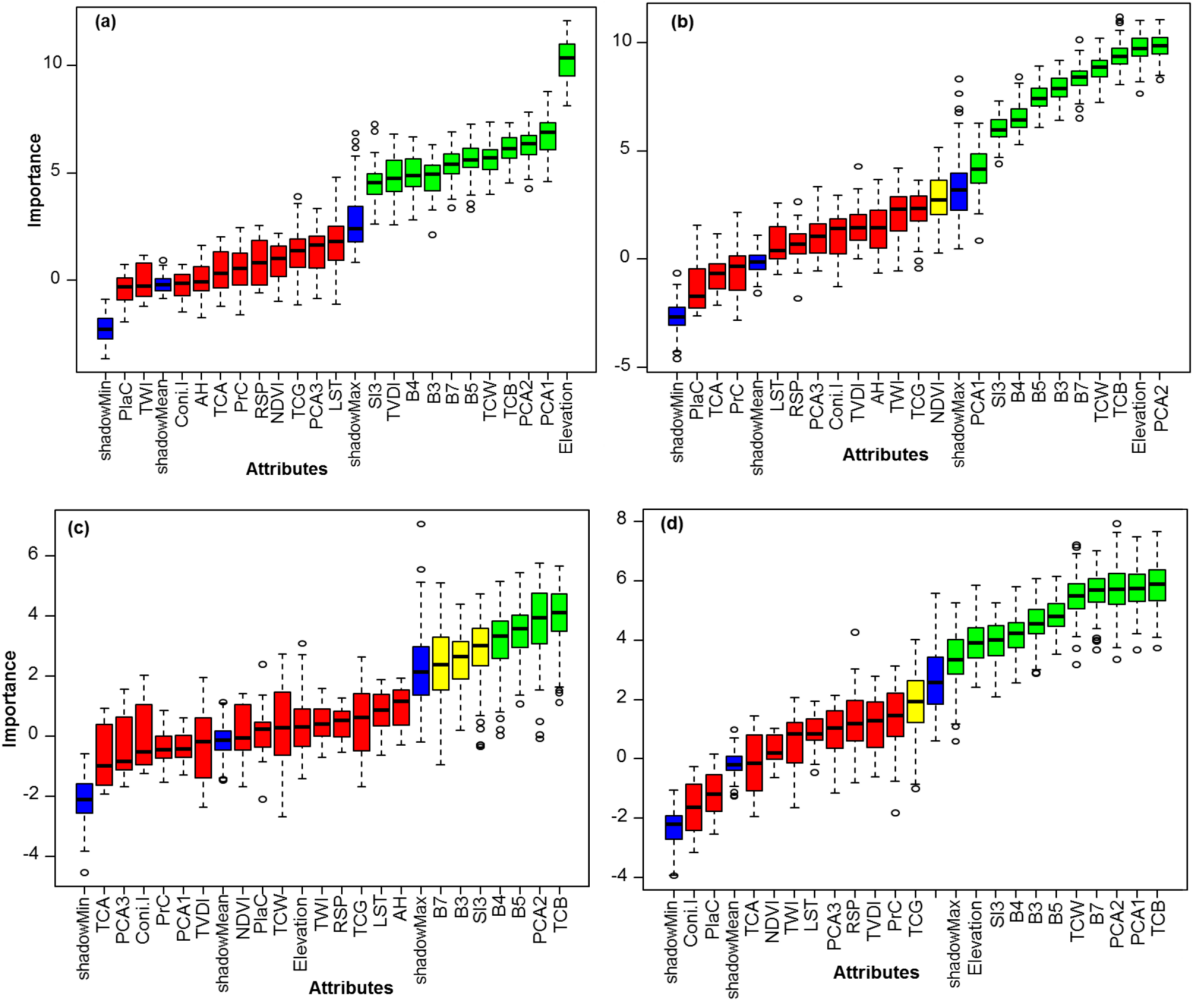
Variable importance determined by the Boruta algorithm for the prediction of (**a**) Ca, (**b**) CaCO_3_, (**c**) CaSO_4_, and (**d**) SO_4_. Variables highlighted in green were identified as important based on the Boruta algorithm’s significance threshold.

The Boruta algorithm was individually applied to each of the four soil attributes, resulting in the selection of between 4 and 11 auxiliary variables, depending on the unique characteristics of each attribute. For calcium (Ca), the Boruta algorithm identified 11 significant predictors, including spectral indices and topographic features (Fig. 4a). In the case of calcium carbonate (CaCO_3_), 9 variables were selected as important, with several bands from satellite imagery and elevation contributing prominently (Fig. 4b). For calcium sulfate (CaSO_4_), 6 influential variables were identified (Fig. 4c), whereas sulfate (SO_4_) was associated with 4 key predictors (Fig. 4d).

**Fig. 5:**
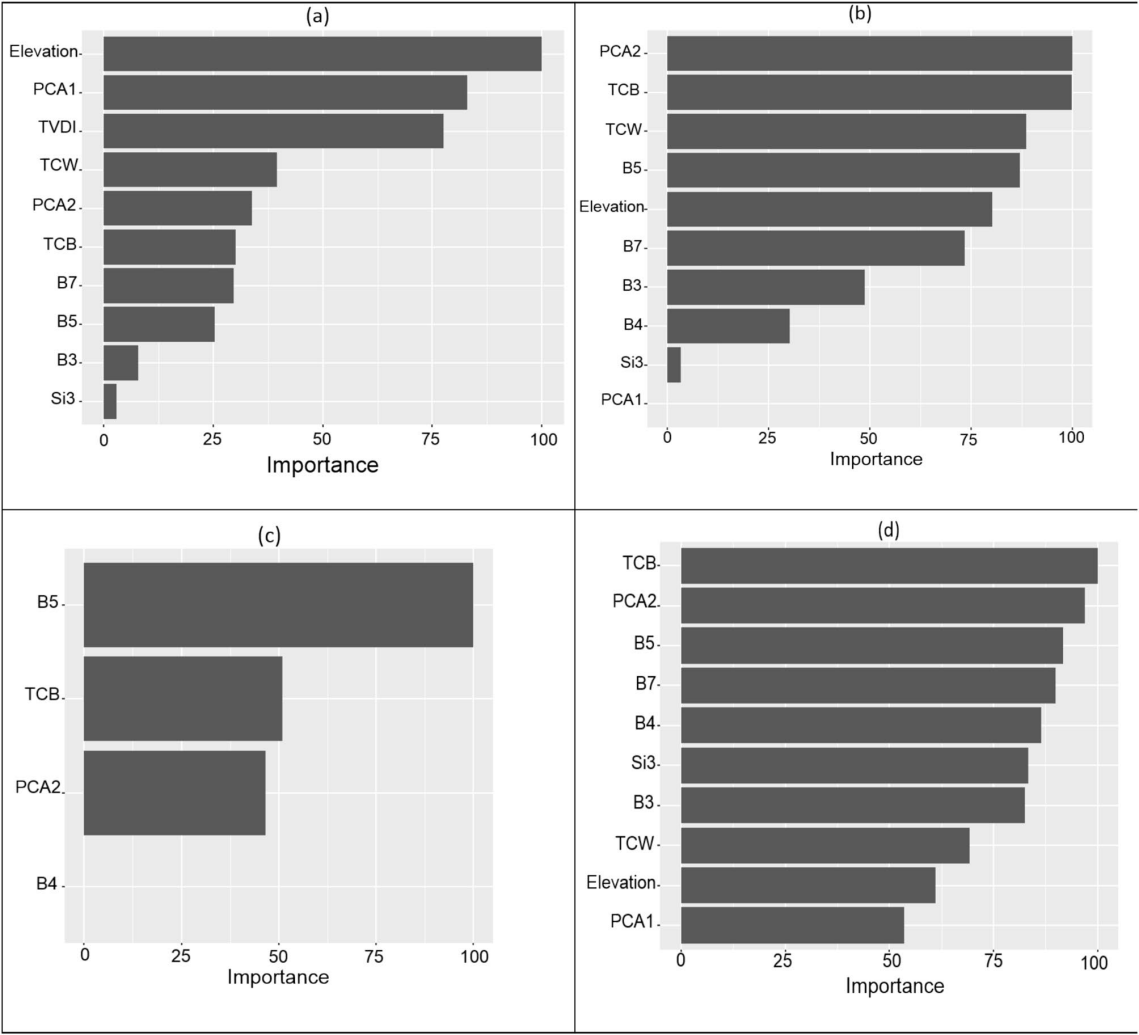
Environmental covariates importance for (**a**) Ca, (**b**) CaCO3, (**c**) CaSO4 and (**d**) SO4 prediction.

Figure 5 highlights the importance of the variables employed in predicting soil properties, with the ensemble model emerging as the top-performing method. The analysis of variable importance identified elevation, PCA1, and TVDI as the most influential factors in predicting soil calcium (Ca). Furthermore, the ensemble model revealed that PCA2 and TCB were the key predictors for determining the distribution of soil calcium carbonate (CaCO_3_) in the study area. For modeling the distribution of calcium sulfate (CaSO_4_), the most important variables were ranked as B5, TCB, and PCA2, respectively. In predicting soil sulfate (SO4), the indices TCB, PCA2, B5, and B7 emerged as the most significant auxiliary variables.

### Comparison of model performance

The performance comparison of different models for predicting soil properties in Table 2 highlights that the ensemble model consistently outperformed all individual models across all metrics (R^2^, RMSE, and MAE). For calcium (Ca), the ensemble model achieved the highest R^2^ value of 0.89, followed by Random Forest (RF) with 0.82, while other models, like GLM and SVR, lagged with R^2^ values of 0.39 and 0.49, respectively. Similarly, the ensemble model also showed the best performance in predicting calcium carbonate (CaCO_3_), calcium sulfate (CaSO_4_), and sulfate (SO_4_), with R^2^ values of 0.84, 0.73, and 0.79, respectively. The ensemble model had the lowest RMSE and MAE values for all soil properties, further indicating its superior predictive accuracy. In particular, for Ca and SO_4_, the ensemble model yielded the lowest RMSE values of 13.69 and 48.37, along with MAE values of 8.15 and 20.93, underscoring its robustness. Although RF performed well, especially for CaSO_4_ with an R^2^ of 0.73, the ensemble model still provided the most accurate predictions across all soil properties.

**Table 2:** Performance of different models for prediction of soil properties.

	Soil properties			
R^2^	Ca	CaCO_3_	CaSO_4_	SO_4_
GLM	0.39	0.59	0.31	0.37
GAM	0.46	0.52	0.32	0.40
CART	0.61	0.61	0.38	0.50
RF	0.82	0.66	0.73	0.51
SVM	0.49	0.59	0.34	0.34
Ensemble	**0.89**	**0.84**	**0.73**	**0.79**
RMSE				
GLM	27.95	6.74	3.66	57.54
GAM	26.41	7.32	3.71	55.37
CART	22.53	6.18	3.99	61.18
RF	16.58	6.27	3.08	49.38
SVM	28.46	6.70	3.24	53.17
Ensemble	**13.69**	**4.06**	**3.08**	**48.37**
MAE				
GLM	19	5.40	1.99	39.70
GAM	17.18	5.86	1.98	38.54
CART	13.90	4.96	2.14	40.91
RF	10.13	4.95	0.91	30.86
SVM	13.35	4.98	1.57	29.90
Ensemble	**8.15**	**2.95**	**0.91**	**20.93**

Significant values are in [bold].

Table 3 lists the optimized hyperparameters for the machine learning models applied to predict soil properties. For both Generalized Linear Models (GLM) and Generalized Additive Models (GAM), the “Gaussian” family was chosen for predicting all soil properties, including Ca, CaCO_3_, CaSO_4_, and SO_4_. In the CART model, the optimal minsplit value was consistently 20, while the complexity parameter (cp) varied, with Ca and SO_4_ using 0.06 and 0.13, respectively, while CaCO_3_ and CaSO_4_ used a cp value of 0. The Random Forest (RF) model was tuned with n_tree_ = 500 for all properties, and the m_try_ values ranged from 2 for CaCO_3_ to 11 for SO_4_. For Support Vector Regression (SVR), the best Sigma and Cost parameters varied across soil properties, with CaSO_4_ requiring the highest Sigma value (1.87) and a Cost of 0.25, while other properties had lower values, such as Ca with Sigma = 0.26 and Cost = 0.5.

**Table 3:** Tuned hyper-parameters of the various machine-learning methods for predicting soil physical and chemical based on the best model.

Soil properties	GLM	GAM		CART		RF		SVM	
	Family	Family	df (1 − 3)	minspilt	Cp (0–0.10)	mtry	ntree	Sigma	C (0.25, 0.50, 1)
Ca	Gaussian	Gaussian	3	20	0.06	7	500	0.26	0.50
CaCO_3_	Gaussian	Gaussian	1	20	0.00	2	500	0.39	1.00
CaSO_4_	Gaussian	Gaussian	2	20	0.00	3	500	1.87	0.25
SO_4_	Gaussian	Gaussian	1	20	0.13	11	500	0.22	0.50

${\text{m}}_{\text{try}}$, No. of variables tried at each split; ${\text{n}}_{\text{tree}}$, Number of trees; minsplit, minimum observation in a node for a split to be attempted; cp, complexity parameter.

### Soil digital maps

**Fig. 6:**
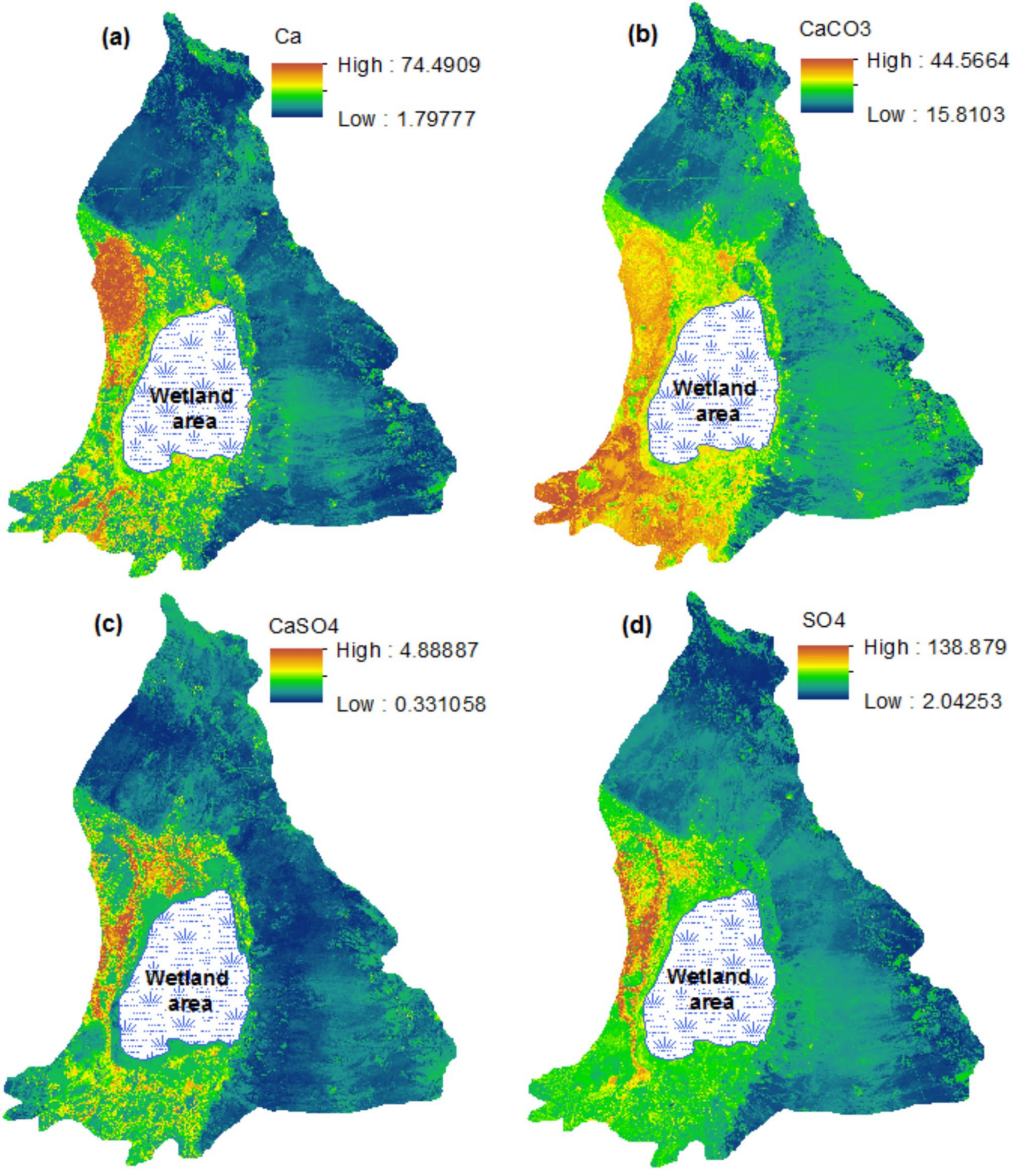
Spatial prediction maps of some soil properties by the best prediction model (Ensemble model) for (**a**) Ca, (**b**) CaCO_3_, (**c**) CaSO_4_ and (**d**) SO_4_ prediction.

Soil property maps were generated using the optimal Ensemble model for each soil parameter. As shown in Fig. 6a, soil calcium (Ca) levels in the study area range from 1.79 to 74.49 meq/L. The northern, eastern, and southeastern edges exhibit lower calcium concentrations, while the western (sand dune) and southwestern regions are characterized by higher calcium levels. Similarly, the percentage of calcium carbonate (CaCO_3_) is elevated in the western and southwestern areas compared to other parts of the region. This pattern is consistent for calcium sulfate (CaSO_4_), with CaCO_3_ values ranging from 15.81 to 44.57 (Fig. 6b) and CaSO_4_ between 0.33 and 4.88 (Fig. 6c). The sulfate (SO_4_) content shows considerable variability, with values spanning from 2.04 to 138.88, highlighting significant spatial variation. The greatest concentrations of SO_4_ are observed in the sand dunes located in the western region, with notable levels also present in the southwestern part of the study area (Fig. 6d).

## Discussion

### Influence of environmental variables and remote sensing data

This study demonstrates the critical role of remote sensing data in improving the prediction of soil properties across a heterogeneous landscape. By incorporating indices a combination of spectral indices and environmental predictors**,** such as TCB, Landsat bands (B7, B5, B3, and B4), and the Salinity Index (SI3), we identified strong correlations between these remote sensing-derived variables and soil properties like calcium carbonate (CaCO_3_) and sulfate (SO_4_).

These results support the hypothesis that spectral reflectance, especially in the SWIR region, is closely linked to surface soil chemistry in arid regions. The positive correlations observed between CaCO_3_ and remote sensing indices reflect the influence of soil composition on surface reflectance properties. We further emphasize that this correlation is not only due to mineral content but also affected by surface conditions, which amplifies spectral brightness.

High levels of CaCO_3_ and SO_4_ tend to increase surface brightness, particularly in arid and semi-arid regions where bare soil or sparse vegetation dominates the landscape. This finding underscores the value of remote sensing in capturing these soil-environment interactions, aligning with previous studies that highlight the utility of remote sensing in digital soil mapping^14,15,63^.

Calcium carbonate (CaCO_3_) and sulfate (SO_4_) are bright minerals that significantly influence the reflectance of soil surfaces in these regions. Their presence enhances the brightness captured by remote sensing indices like TCB and SI3, which are reliable proxies for carbonate and sulfate concentrations. Landsat bands, particularly those in the shortwave infrared (SWIR) region such as B5 and B7, are particularly sensitive to the reflectance of these minerals. The absorption features within these bands align with the presence of CaCO_3_ and SO_4_, enabling precise detection and mapping of these minerals across the landscape. As a result, the high reflectance of CaCO_3_ and SO_4_ in these bands correlates strongly with the spectral signature captured by remote sensing tools^64,65^.

Furthermore, the identification of key predictors such as elevation, PCA1, and TVDI for soil calcium, and PCA2 and TCB for calcium carbonate, provides valuable insights into the environmental factors that govern soil variability. Elevation influences soil calcium distribution by shaping microclimatic conditions (e.g., precipitation and temperature) and controlling runoff and sediment deposition patterns. In low-elevation areas, depositional environments are more likely to accumulate calcium-rich sediments, whereas high-elevation zones may experience leaching due to increased rainfall. Consistent with these findings, altitude, along with annual mean rainfall and the Normalized Difference Salinity Index (NDSI), were among the most influential predictors for soil pH distribution in arid and semi-arid landscapes using Random Forest modeling^21^. These patterns have been corroborated in studies exploring topographic controls on soil nutrients^66,67^.

TVDI reflects moisture availability and vegetation stress, which directly influence soil properties in arid and semi-arid conditions. Soils in drier regions with stressed vegetation are often characterized by increased carbonate and sulfate concentrations due to reduced leaching and enhanced deposition of weathered materials. The strong correlation of TCB with calcium carbonate highlights its utility in detecting bright, carbonate-rich soils, particularly in landscapes dominated by bare surfaces^6,68^.

In regions with minimal vegetation cover, such as those dominated by bare soil, the spectral signal is primarily influenced by soil properties rather than vegetation. This helps explain the strong correlation between remote sensing indices and soil chemical properties observed in our study. Furthermore, surface crusting, which is common in soils rich in carbonates and sulfates, amplifies reflectance by reducing soil roughness and enhancing surface brightness. These characteristics make remote sensing indices even more effective in detecting and mapping soil properties in such environments.

These findings underscore the value of remote sensing data in capturing soil-environment interactions and improving the spatial resolution of soil property maps. By providing spatially extensive, consistent information, remote sensing data enhance the precision of digital soil mapping, supporting informed decision-making for sustainable agricultural practices and soil conservation. This is particularly critical in arid and semi-arid regions, where soil degradation poses significant challenges to land management and food security.

Moreover, the enhanced detection of CaCO_3_ using TCB and PCA2 underscores the power of principal component transformation in reducing dimensionality and isolating meaningful spectral patterns associated with specific soil constituents. These results are consistent with recent advances in digital soil mapping literature and confirm the potential of integrating spectral indices, topographic variables, and principal components to improve prediction accuracy in dryland regions^69^.

### Effectiveness of machine learning models

The integration of remote sensing data with machine learning algorithms has become a transformative approach in digital soil mapping, especially for predicting soil properties in complex and heterogeneous landscapes. In this study, among all tested models, the Ensemble model consistently outperformed others, delivering the highest R^2^ and the lowest RMSE and MAE across key soil properties such as calcium, calcium carbonate, calcium sulfate, and sulfate. The superior performance of the Ensemble model stems from its ability to aggregate predictions from diverse base learners, balancing individual model biases and reducing variance caused by overfitting. This synergy enables the model to capture both broad trends and localized variations in soil characteristics, resulting in more robust and accurate predictions^70,71^.

In contrast, traditional regression-based methods such as GLM and GAM showed limited performance. Their reliance on linear or semi-linear assumptions restricts their capacity to model complex, nonlinear interactions between soil properties and environmental predictors. For instance, relationships involving RS indices like TCB and SI3 often include threshold effects, interactions, and saturation points, dynamics poorly captured by linear approaches^25^. This limitation was reflected in their relatively low R^2^ values (e.g., 0.39 for Ca in GLM and 0.46 in GAM) and higher RMSE values, suggesting reduced suitability for DSM in heterogeneous regions.

Nonlinear models such as RF, CART, and SVR outperformed GLM and GAM, indicating their effectiveness in handling complex interactions and high-dimensional data. Among these, RF emerged as particularly powerful, achieving an R^2^ of 0.82 for Ca and maintaining consistently low RMSE and MAE scores. RF’s ensemble-based architecture, which combines multiple decision trees, helps minimize overfitting and improves generalization, while also offering insights into variable importance^72,73^. CART, though slightly less accurate than RF, remained valuable due to its interpretability and ability to highlight dominant predictors of soil variability.

SVR showed moderate performance relative to RF and the Ensemble model. While SVR is adept at managing high-dimensional spaces and avoids overfitting, its effectiveness is contingent on careful kernel and parameter tuning. Without optimal settings, SVR may underperform, as suggested by its lower R^2^ values and higher errors in this study^74^. Future research could enhance SVR performance by refining hyperparameters or integrating it within hybrid modeling frameworks.

The clear outperformance of the Ensemble model across all metrics underscores the advantage of model stacking and blending in soil prediction tasks. For example, the Ensemble model reached an R^2^ of 0.89 for Ca, notably higher than RF (R^2^ = 0.82) and SVR (R^2^ = 0.49), while also yielding the lowest RMSE and MAE values. These results reflect the model’s ability to combine the predictive strength of RF with the interpretability of simpler algorithms like CART, leading to a more balanced and resilient predictive system.

The variation in model performance highlights the critical importance of algorithm selection in environmental modeling. Soil properties are shaped by a complex set of biophysical factors including mineral composition, moisture availability, and topographic features that often interact in nonlinear and spatially variable ways. Ensemble methods and nonlinear models like RF are better equipped to capture such relationships, as supported by numerous DSM studies^72,75^. Furthermore, the use of RS data enhances the models’ predictive capacity by offering spatially detailed information on environmental processes.

These findings demonstrate the potential of advanced ML models, particularly in arid and semi-arid regions where sparse data and high environmental variability are major challenges^76^. The ability of RF and Ensemble models to handle such complexity, combined with the broad spatial reach of RS data, makes them indispensable tools for precision agriculture and sustainable land management.

Notably, nonlinear models such as RF and CART also provide valuable insights into variable importance, helping to identify key environmental drivers of soil properties^77^. This interpretability, along with high predictive accuracy, reinforces the growing consensus on the effectiveness of ensemble approaches in heterogeneous and data-limited settings.

It is also important to contextualize model error metrics. In this study, the mean observed value of Ca was 25.68 meq/L. The Ensemble model achieved an RMSE of 13.69 and an MAE of 8.15, values well below the mean, indicating high predictive accuracy. Similar patterns were observed for other soil variables, further supporting the robustness of the Ensemble model. Generally, RMSE and MAE values approaching zero signify lower prediction error and improved model performance.

### Implications for soil management

The spatial patterns of soil properties revealed by this study have important implications for soil management, particularly in regions like the western sand dunes, where significant variations in calcium, carbonate, sulfate, and sulfate concentrations were observed. These variations are heavily influenced by local environmental conditions. They underscore the need for tailored, localized soil management strategies. The Ensemble model’s ability to generate accurate soil property maps provides valuable information that can guide environmental interventions aimed at mitigating soil degradation and improving land productivity.

In areas with elevated levels of lime and gypsum, such as the western and southwestern regions of the study area, these soil characteristics are primarily driven by the underlying parent materials, including limestone and marl. The presence of evaporite minerals like calcite and gypsum is also influenced by the high groundwater table around the salt marsh. In Iran’s arid and semi-arid regions, where soils typically exhibit high pH values (greater than 7) and abundant calcium carbonate, these conditions can hinder plant growth by limiting the availability of nutrients such as phosphorus, iron, and zinc^78,79^. This further highlights the importance of accurate soil property mapping for improving agricultural practices and ensuring long-term environmental sustainability.

The heterogeneity of the study area’s geomorphological surfaces, which include high and medium elevations, sand dunes, gypsum plateaus, lowlands with high gravel proportions, glaciated areas, wetlands, playas surrounding the wetlands, floodplains, and a delta, adds to the complexity of soil property distribution. This diversity in landforms creates varied conditions for soil formation, contributing to the significant differences observed in soil composition and structure. The use of remote sensing data, combined with advanced machine learning models, offers a robust solution for capturing and predicting these intricate patterns of soil variability. Traditional methods may fail to account for this complexity, but the detailed maps produced through this study provide essential insights for managing diverse landscapes.

### Study limitations and future research opportunities

Although the findings of this research are encouraging, several limitations should be acknowledged to guide future investigations. A key consideration is the use of climatic data. In this study, direct climatic variables such as precipitation and air temperature were not included. This decision was based on the relatively small size of the watershed, the limited climatic variability across the area, and the presence of only one climatological station. These factors reduced the feasibility and potential value of integrating coarse-resolution climatic datasets.

Nevertheless, relevant climate-related information was still incorporated through remote sensing–based indices such as LST and TVDI. These indices effectively captured spatial variations in surface temperature and drought stress, which are important drivers of soil processes in arid and semi-arid regions.

Furthermore, due to the extremely low and relatively uniform annual rainfall, which is generally less than 80 mm, and the strong influence of geomorphological and soil-forming factors such as parent material, salinity, and groundwater levels, indirect climate proxies were considered sufficient to address the study’s objectives.

However, for larger or more climatically diverse regions, future research should consider the integration of gridded climatic datasets such as CHELSA bioclimatic variables or downscaled data from meteorological stations. Including such data could potentially improve the accuracy and generalizability of soil prediction models.

Additionally, increasing the number of soil samples and the range of auxiliary variables could enhance the precision of spatial distribution models. A more extensive dataset would allow for a more comprehensive representation of the environmental factors that influence soil properties. This, in turn, would strengthen the predictive capacity of machine learning models. Improved modeling of soil variability has important implications for sustainable land management, especially in areas facing challenges such as soil degradation, water scarcity, and reduced agricultural productivity.

## Conclusion

This study demonstrates the effectiveness of integrating remote sensing data with advanced machine learning techniques for predicting key soil properties in arid and semi-arid landscapes. By leveraging spectral indices, topographic variables, and principal components, we achieved high predictive accuracy for calcium, calcium carbonate, and sulfate contents. The Ensemble model, in particular, outperformed other algorithms and provided robust predictions across multiple soil attributes. These findings affirm the transformative potential of ensemble-based digital soil mapping, especially in data-scarce and environmentally heterogeneous regions. The novelty of this work lies in its combination of dimensionality reducing techniques such as PCA with multiple remote sensing indicators in an ensemble learning framework. This integration enhances prediction performance over traditional and standalone models. Our approach captures complex nonlinear soil-environment interactions and enables spatially detailed, scalable mapping that is critical for resource planning. Beyond methodological contributions, this research provides actionable insights for sustainable soil management. Accurate soil property maps, as generated here, can inform land use planning, guide site-specific agricultural inputs, and support reclamation strategies in degraded drylands. These outputs are particularly relevant in the context of national efforts to combat desertification and optimize water and soil resources. While we acknowledge inherent limitations such as the influence of surface roughness or vegetation cover on reflectance, our results support the broader application of remote sensing combined with ensemble modeling in digital soil mapping. Future research should explore temporal monitoring using time series data, integration of hyperspectral imagery, and model adaptation across different eco-regions. Given the pressing challenges posed by climate change and soil degradation in arid zones, we recommend that policymakers incorporate digital soil maps into regional land management frameworks. Aligning such data-driven approaches with environmental regulations such as Iran’s soil conservation strategies and land rehabilitation programs can enhance policy effectiveness and ecological outcomes.

## Figures and Tables

**Fig. 7:**
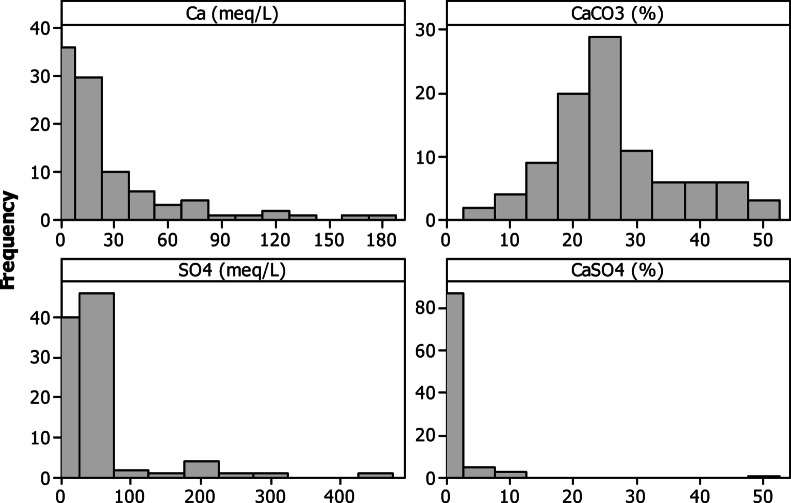
Frequency histograms illustrating the distribution of soil chemical properties including calcium (Ca), calcium carbonate (CaCO_3_), calcium sulfate (CaSO_4_), and sulfate (SO_4_) across the study area.

## Data Availability

The data that support the findings of this study are available from the corresponding author upon reasonable request. The data are not publicly available because of privacy or ethical restrictions.

## Appendix 1

See Table 4

**Table 4:** Environmental covariates derived from a 30-m spatial resolution Landsat imagery.

Environmental covariate	Formula
Landsat 8 OLI bands and thermal band	B2—Blue (0.450–0.51 µm) with 30 m resolution
	**B3—**Green (0.53–0.59 µm) with 30 m resolution
	**B4—**Red (0.64–0.67 µm) with 30 m resolution
	**B5—**Near-Infrared (0.85–0.88 µm) with 30 m resolution
	B6—SWIR1 (1.57–1.65 µm) with 30 m resolution
	**B7**—SWIR2 (2.11–2.29 µm) with 30 m resolution
Principal component analysis (PCA)	**PCA1**
	**PCA2**
	**PCA3**
Tasseled cap brightness (TCB)	**TCB** = (Blue × 0.3029) + (Green × 0.2786) + (Red × 0.4733) + (NIR × 0.5599) + (SWIR1 × 0.508) + (SWIR2 × 0.1872)
Tasseled cap greenness (TCG)	**TCG** = Blue × (0.2941) + Green × (0.243) + Red × (0.5424) + NIR × 0.7276 + SWIR1 × 0.0713 + SWIR2 × (‒0.1608)
Tasseled cap wetness (TCW)	**TCW** = Blue × 0.1511 + Green × 0.1973 + Red × 0.3283 + NIR × 0.3407 + SWIR1 × (‒0.7117) + SWIR2 × (‒0.4559)
Soil brightness index (BI)	$\text{BI}=\sqrt{{\text{Red}}^{2}+{\text{NIR}}^{2}}$
Soil salinity index 1 (SI1)	$\text{SI}1=\sqrt{\text{Green}\times \text{Red}}$
Soil salinity index 2 (SI2)	$\text{SI}2=\sqrt{{\text{Green}}^{2}+{\text{Red}}^{2}+{\text{NIR}}^{2}}$
Soil salinity index 3 (SI3)	$\mathbf{S}\mathbf{I}3=\sqrt{{\text{Green}}^{2}+{\text{Red}}^{2}}$
Modified soil-adjusted vegetation index (MSAVI)	$\text{MSAVI}=\frac{2\text{ NIR}+1-\sqrt{{(2\text{ NIR}+1)}^{2}-8(\text{NIR}-\text{Red})}}{2}$
Normalized difference vegetation index (NDVI)	${\mathbf{NDVI}} = \frac{{\left( {{\text{NIR}} - {\text{Red}}} \right)}}{{\left( {{\text{NIR}} + {\text{Red}}} \right)}}$
Land surface temperature (LST) (°C)	**LST** = image × (Thermal Infrared) × (0.00341802) + (149)
Temperature vegetation drought index (TVDI)	$\mathbf{T}\mathbf{V}\mathbf{D}\mathbf{I}=\frac{\text{LST}-{\text{LST}}_{\text{min}}}{{\text{LST}}_{\text{max}}-{\text{LST}}_{\text{min}}}$

Variables highlighted in bold indicate those used in the modeling process.

.

## Appendix 2

See Table 5

**Table 5:** Terrain attributes derived from digital elevation model (DEM).

Parameter	Definition
DEM (Elevation)- 30 m	Represents the vertical height above mean sea level, crucial for analyzing soil erosion, sediment dynamics, and overall landform structure
Analytical hillshading (AH)	Assesses the angle between the terrain surface and incoming light, enhancing the visualization of terrain features and their impact on soil processes
Aspect	Defines the compass direction of the steepest slope, influencing factors such as solar exposure and patterns of water runoff
Channel network base level (CNBL)	Provides interpolated elevations of channel networks, helping to determine landscape lowering and the effectiveness of drainage systems
Channel network distance (CND)	Measures the distance from any given point to the nearest channel network, offering insights into spatial connectivity and its effects on water and sediment flow
Convergence index (Conv.I)	Derived from aspect data, this index reveals how terrain relief affects the convergence of water flow, critical for understanding water movement and distribution
Plan curvature (PlaC) (Degrees)	Indicates the curvature of the terrain when intersected with a horizontal plane, impacting water flow, erosion, and deposition processes
Profile curvature (PrC)	Evaluates the rate of change in slope along the flow direction, which differentiates between areas of erosion and deposition
Relative slope position (RSP)	Measures the relative distance from the base of a slope to the nearest ridge, influencing drainage, erosion patterns, and local environmental conditions
Slope (Degrees)	Describes the average gradient along the flow path, affecting processes like soil erosion, sediment transport, and water movement
Topographic wetness index (TWI)	Assesses how topography influences soil moisture, providing insights into hydrological dynamics and soil water content
Total catchment area (TCA)	Estimates the volume of runoff expected, essential for understanding patterns of water inflow and sediment movement within the catchment
Length slope factor (LS_F)	Measures the volume of surface flow, which impacts soil erosion and sediment transport within a catchment area
Valley depth (VD)	Determines soil characteristics by assessing the depth of valleys, which affects soil composition, fertility, and land management strategies

Variables highlighted in bold indicate those used in the modeling process.

.

## Appendix 3

See Fig. 7

.

## Abbreviations

AH: Analytical hillshading
BI: Soil brightness index
Ca: Calcium
CaCO_3_: Calcium carbonate
CaSO_4_: Calcium sulfate
CART: Classification and regression trees
CNBL: Channel network base level
CND: Channel network distance
Conv.I: Convergence index
CV: Coefficient of variation
DEM: Digital elevation model (Elevation)-30 m
GAM: Generalized additive model
GLM: Generalized linear model
LS_F: Length slope factor
LST: Land surface temperature (°C)
MAE: Mean absolute error
MSAVI: Modified soil-adjusted vegetation index
ML: Machine learning
MSAVI: Modified soil-adjusted vegetation index
NDVI: Normalized difference vegetation index
PCA: Principal component analysis
PCA1: First principal component
PCA2: Second principal component
PlaC: Plan curvature (Degrees)
PrC: Profile curvature
RF: Random forest
R^2^: Coefficient of determination
RBF: Radial basis function
RMSE: Root mean square error
RSP: Relative slope position
RS: Remote sensing
SI1: Soil salinity index 1
SI2: Soil salinity index 2
SI3: Soil salinity index 3
SOC: Soil organic carbon
SO_4_: Sulfate
SRTM: Shuttle radar topography mission
SVR: Support vector regression
TCA: Total catchment area
TCB: Tasseled cap brightness
TCG: Tasseled cap greenness
TCW: Tasseled cap wetness
TCT: Tasseled cap transformation
TVDI: Temperature vegetation drought index
TWI: Topographic wetness index
usdm: Uncertainty analysis for species distribution models (R package)
VD: Valley depth
varImp: Variable importance (Function in R caret package)
VIF: Variance inflation factor

## References

[1] Rutgers, M. et al. Mapping soil biodiversity in Europe and The Netherlands. *Soil Syst.* **3**(2), 39 (2019).

[2] Barrena-González, J., Lavado Contador, F., Repe, B. & Pulido Fernández, M. Looking for optimal maps of soil properties at the regional scale. *Int. J. Environ. Res.* **18**(4), 1–22 (2024).

[3] Naeij Nouri, R. Investigation of the possibility of differentiating saline and gypsum lands in the Kashan Plain using TM satellite data. In *Master’s Thesis (Desertification)*, Isfahan University of Technology, Faculty of Natural Resources, Iran, 125 (In Persian) (2001).

[4] Forkuor, G., Hounkpatin, O. K., Welp, G. & Thiel, M. High resolution mapping of soil properties using remote sensing variables in south-western Burkina Faso: A comparison of machine learning and multiple linear regression models. *PLoS ONE* **12**(1), e0170478 (2017). 28114334 10.1371/journal.pone.0170478 PMC5256943

[5] McBratney, A. B., Santos, M. M. & Minasny, B. On digital soil mapping. *Geoderma* **117**(1–2), 3–52 (2003).

[6] Mulder, V. L., De Bruin, S., Schaepman, M. E. & Mayr, T. R. The use of remote sensing in soil and terrain mapping—A review. *Geoderma* **162**(1–2), 1–19 (2011).

[7] Norby, J. et al. Path to autonomous soil sampling and analysis by ground-based robots. *J. Environ. Manag.* **360**, 121130 (2024). 10.1016/j.jenvman.2024.121130 38772232

[8] Wang, J. et al. Remote sensing of soil degradation: Progress and perspective. *Int. Soil Water Conserv. Res.* **11**(3), 429–454 (2023).

[9] Jenny, H. *Factors of Soil Formation: A System of Quantitative Pedology* 281 (McGraw Hill, 1941).

[10] Alemayehu, B., Suarez-Minguez, J. & Rosette, J. Modeling the spatial distribution of *Acacia decurrens* plantation forests using planetscope images and environmental variables in the Northwestern Highlands of Ethiopia. *Forests* **15**(2), 277 (2024).

[11] Fathololoumi, S. et al. Improved digital soil mapping with multitemporal remotely sensed satellite data fusion: A case study in Iran. *Sci. Total Environ.* **721**, 137703 (2020). 32172111 10.1016/j.scitotenv.2020.137703

[12] Rahmanian, S. et al. Prediction of plant diversity using multi-seasonal remotely sensed and geodiversity data in a mountainous area. *Remote Sens.* **15**(2), 387 (2023).

[13] Abdulraheem, M. I. et al. Advancement of remote sensing for soil measurements and applications: A comprehensive review. *Sustainability* **15**(21), 15444 (2023).

[14] Barnes, E. M. & Baker, M. G. Multispectral data for mapping soil texture: Possibilities and limitations. *Appl. Eng. Agric.* **16**(6), 731–741 (2000).

[15] Castaldi, F. et al. Evaluation of the potential of the current and forthcoming multispectral and hyperspectral imagers to estimate soil texture and organic carbon. *Remote Sens. Environ.* **179**, 54–65 (2016).

[16] Sayão, V. M., Demattê, J. A., Bedin, L. G., Nanni, M. R. & Rizzo, R. Satellite land surface temperature and reflectance related with soil attributes. *Geoderma* **325**, 125–140 (2018).

[17] Faramarzi, S. E., Pazira, E., Masihabadi, M. H., Mohammadi Torkashvand, A. & Motamedvaziri, B. Modeling and estimating the spatial distribution of soil organic matter content in irrigated lands. *Int. J. Environ. Sci. Technol.* **19**, 7399–7410 (2022).

[18] Khosravi Aqdam, K., Asadzadeh, F., Rezapour, S. & Nouri, A. Comparative assessment of soil fertility across varying elevations. *Environ. Monit. Assess.* **195**, 1007 (2023). 10.1007/s10661-023-11610-1 37515672

[19] Ghavami, M. S., Ayoubi, S., Mosaddeghi, M. R. & Naimi, S. Digital mapping of soil physical and mechanical properties using machine learning at the watershed scale. *J. Mt. Sci.* **20**(10), 2975–2992 (2023).

[20] Bao, Y. et al. A fine digital soil mapping by integrating remote sensing-based process model and deep learning method in Northeast China. *Soil Tillage Res.* **238**, 106010 (2024).

[21] Amindin, A., Siamian, N., Ahmadi, F., Kariminejad, N. & Pourghasemi, H. R. Digital mapping of soil pH in arid and semi-arid regions. In *Advanced Tools for Studying Soil Erosion Processes* (eds. Rodrigo-Comino, J. & Brevik, E. C.) 485–501 (Elsevier, 2024).

[22] Franklin, J. *Mapping Species Distributions: Spatial Inference and Prediction* (Cambridge University Press, 2010).

[23] Hastie, T. & Tibshirani, R. *Generalized Additive Models* 1 (Chapman and Hall/CRC, 1990).

[24] Breiman, L. Random forests. *Mach. Learn.* **45**(1), 5–32 (2001).

[25] Hengl, T. et al. Mapping soil properties of Africa at 250 m resolution: Random forests significantly improve current predictions. *PLoS ONE* **10**(6), e0125814 (2015). 26110833 10.1371/journal.pone.0125814 PMC4482144

[26] Ramsar, I. Convention on wetlands of international importance, especially as waterfowl habitat. Ramsar (Iran). (1971).

[27] Jafari, R. & Hasheminasab, S. Assessing the effects of dam building on land degradation in central Iran with Landsat LST and LULC time series. *Environ. Monit. Assess.* **189**, 1–15 (2017). 10.1007/s10661-017-5792-y 28116607

[28] Pirali Zefrehei, A. R., Kolahi, M. & Fisher, J. Modeling wetland restoration scenarios in Gavkhooni International Wetland. *Restor. Ecol.* **31**, e13721 (2023).

[29] Rai, A. K., Basak, N. & Sundha, P. Chemistry of salt-affected soils. In *Managing Salt-Affected Soils for Sustainable Agriculture* (eds Minhas, P. C. S. P. S. & Yadav, R. K.) 128–148 (Indian Council of Agricultural Research ICAR, 2021).

[30] Bashari, H. *Development of an evaluation system to assess structural and functional vegetation changes in the Gavkhouni International Wetland region* (Environmental Protection Agency of Isfahan Province, 2022) (**In Persian**).

[31] Soil Survey Staff. Keys to soil taxonomy. (United States Department of Agriculture, Washington, DC, 2014).

[32] Carter, M. R. & Gregorich, E. G. *Soil Sampling Methods of Analysis* 2nd edn, 1224 (CRC Press, 2008).

[33] Baig, M. H. A., Zhang, L., Shuai, T. & Tong, Q. Derivation of a tasselled cap transformation based on Landsat 8 at-satellite reflectance. *Remote Sens. Lett.* **5**(5), 423–431 (2014).

[34] Allbed, A. & Kumar, L. Soil salinity mapping and monitoring in arid and semi-arid regions using remote sensing technology: A review. *Adv. Remote Sens.* **2**(4), 373–385 (2013).

[35] Jolliffe, I. T. *Principal Component Analysis for Special Types of Data* 338–372 (Springer, 2002).

[36] Khan, N. M., Rastoskuev, V. V., Shalina, E. V. & Sato, Y. Mapping salt-affected soils using remote sensing indicators—a simple approach with the use of GIS IDRISI. In *22nd Asian Conference on Remote Sensing* **5**, (9). (2001).

[37] Douaoui, A. & Lepinard, P. Remote sensing & soil salinity: Mapping of soil salinity in the Algerian plain-Lower-Cheliff. *Geomat. Expert* **76**, 36–41 (2010).

[38] Douaoui, A., Nicolas, H. & Walter, C. Detecting salinity hazards within a semiarid context by means of combining soil and remote-sensing data. *Geoderma* **134**(1–2), 217–230 (2006).

[39] Rouse, J. W., Haas, R. H., Schell, J. A. & Deering, D. W. Monitoring vegetation systems in the Great Plains with ERTS. *NASA Spec. Publ.* **351**(1), 309 (1974).

[40] Qi, J., Chehbouni, A., Huete, A. R., Kerr, Y. H. & Sorooshian, S. A modified soil adjusted vegetation index. *Remote Sens. Environ.* **48**(2), 119–126 (1994).

[41] Ermida, S. L., Soares, P., Mantas, V., Göttsche, F. M. & Trigo, I. F. Google earth engine open–source code for land surface temperature estimation from the landsat series. *Remote Sens.* **12**(9), 1471 (2020).

[42] Han, Y., Wang, Y. & Zhao, Y. Estimating soil moisture conditions of the greater Changbai Mountains by land surface temperature and NDVI. *IEEE Trans. Geosci. Remote Sens.* **48**(6), 2509–2515 (2010).

[43] Planchon, O. & Darboux, F. A fast, simple and versatile algorithm to fill the depressions of digital elevation models. *CATENA* **46**(2–3), 159–176 (2002).

[44] Moharana, P. C. et al. Digital soil mapping algorithm for soil quality assessment and monitoring: a case study in desert ecosystem of India. In *Remote Sensing of Soils* 229–245 (Elsevier, 2024).

[45] Hengl, T., Rossiter, D. G. & Stein, A. Soil sampling strategies for spatial prediction by correlation with auxiliary maps. *Soil Res.* **41**(8), 1403–1422 (2003).

[46] Akinwande, M. O., Dikko, H. G. & Samson, A. Variance inflation factor: As a condition for the inclusion of suppressor variable (s) in regression analysis. *Open J. Stat.* **5**(07), 754 (2015).

[47] Khosravani, P., Baghernejad, M., Moosavi, A. A. & Rezaei, M. Digital mapping and spatial modeling of some soil physical and mechanical properties in a semi-arid region of Iran. *Environ. Monit. Assess.* **195**(11), 1367 (2023). 37875717 10.1007/s10661-023-11980-6

[48] Kursa, M. B. & Rudnicki, W. R. Feature selection with the Boruta package. *J. Stat. Softw.* **36**, 1–13 (2010).

[49] O’brien, R. M. A caution regarding rules of thumb for variance inflation factors. *Qual. Quant.* **41**, 673–690 (2007).

[50] Kuhn, M. *Predictive Modeling with R and the Caret Package* (Pfizer Global RandD, 2013).

[51] Guisan, A., Edwards, T. C. Jr. & Hastie, T. Generalized linear and generalized additive models in studies of species distributions: Setting the scene. *Ecol. Model.* **157**(2–3), 89–100 (2002).

[52] Gbur, E. E. et al. *Analysis of generalized linear mixed models in the agricultural and natural resources sciences* Vol. 156 (Wiley, 2020).

[53] Breiman, L., Friedman, J. H., Olshen, R. A. & Stone, C. J. *Classification and Regression Trees* (Chapman and Hall, 1984).

[54] Therneau, T., Atkinson, B. & Ripley, B. Rpart: Recursive Partitioning and Regression Trees. R Package Version 4.1-11. https://CRAN.R-project.org/package=rpart. (2017).

[55] Belgiu, M. & Dragut, L. Random forest in remote sensing: A review of applications and future directions. ISPRS J. Photogramm. Remote Sens.**114**, 24–31 (2016).

[56] Hengl, T. & McMillan, R. A. Predictive soil mapping with R. OpenGeoHub Foundation, Wageningen, The Netherlands, 978-0-359-30635-0. (2019).

[57] Vapnik, V. *The Nature of Statistical Learning Theory* Vol. 10, 978–981 (Springer, 1995).

[58] Deiss, L., Margenot, A. J., Culman, S. W. & Demyan, M. S. Tuning support vector machines regression models improves prediction accuracy of soil properties in MIR spectroscopy. *Geoderma* **365**, 114227 (2020).

[59] Tajik, S., Ayoubi, S. & Zeraatpisheh, M. Digital mapping of soil organic carbon using ensemble-learning model in Mollisols of Hyrcanian forests, northern Iran. *Geoderma Reg.* **20**, e00256 (2020).

[60] Zhou, Z. H. *Ensemble methods: foundations and algorithms* (CRC Press, 2025).

[61] Willmott, C. J. & Matsuura, K. Advantages of the mean absolute error (MAE) over the root mean square error (RMSE) in assessing average model performance. *Climate Res.* **30**(1), 79–82 (2005).

[62] Wilding, L. P. Spatial variability: its documentation, accommodation and implication to soil surveys. In *Soil Spatial Variability*, 166–194 (1985).

[63] Dash, P. K. Remote sensing as a potential tool for advancing digital soil mapping. In *Remote Sensing of Soils* 357–370 (Elsevier, 2024).

[64] Abdulhussein, A. S. A. & Mihalache, M. The use of satellite images for the identification of salinized soils in Brăila county. *Sci. Papers Ser. A Agron.* **65**(2), 11–21 (2022).

[65] Lillesand, T., Kiefer, R. W. & Chipman, J. *Remote Sensing and Image Interpretation* (Wiley, 2015).

[66] Adhikari, K. et al. Digital mapping of soil organic carbon contents and stocks in Denmark. *PLoS ONE* **9**(8), e105519 (2014). 25137066 10.1371/journal.pone.0105519 PMC4138211

[67] Zhou, W., Li, C., Zhao, W., Stringer, L. C. & Fu, B. Spatial distributions of soil nutrients affected by land use, topography and their interactions, in the Loess Plateau of China. *Int. Soil Water Conserv. Res.* **12**(1), 227–239 (2024).

[68] Zhang, J. et al. Spectral analysis of seasonal rock and vegetation changes for detecting karst rocky desertification in southwest China. *Int. J. Appl. Earth Obs. Geoinf.* **100**, 102337 (2021).

[69] Zhao, S. et al. Integrating proximal soil sensing data and environmental variables to enhance the prediction accuracy for soil salinity and sodicity in a region of Xinjiang Province, China. *J. Environ. Manag.* **364**, 121311 (2024). 10.1016/j.jenvman.2024.121311 38875977

[70] Adeniyi, O. D., Brenning, A., Bernini, A., Brenna, S. & Maerker, M. Digital mapping of soil properties using ensemble machine learning approaches in an agricultural lowland area of Lombardy, Italy. *Land* **12**(2), 494 (2023).

[71] Taghizadeh-Mehrjardi, R. et al. Improving the spatial prediction of soil organic carbon content in two contrasting climatic regions by stacking machine-learning models and rescanning covariate space. *Remote Sens.* **12**(7), 1095 (2020).

[72] Camera, C. et al. A high-resolution map of soil types and physical properties for Cyprus: A digital soil mapping optimization. *Geoderma* **285**, 35–49 (2017).

[73] Zeraatpisheh, M., Ayoubi, S., Jafari, A., Tajik, S. & Finke, P. Digital mapping of soil properties using multiple machine learning in a semi-arid region, central Iran. *Geoderma* **338**, 445–452 (2019).

[74] Azizi, K., Garosi, Y., Ayoubi, S. & Tajik, S. Integration of Sentinel-1/2 and topographic attributes to predict the spatial distribution of soil texture fractions in some agricultural soils of western Iran. *Soil Tillage Res.* **229**, 105681 (2023).

[75] John, K. et al. Using machine-learning algorithms to estimate soil organic carbon variability with environmental variables and soil nutrient indicators in an alluvial soil. *Land* **9**(12), 487 (2020).

[76] Gulledmath, S. & Hemanth, K. S. Leveraging machine learning: Advanced algorithms for soil data analysis and feature extraction in arid and semi-arid regions with expert systems. *SN Comput. Sci.* **5**(7), 902 (2024).

[77] Huang, H. et al. Digital mapping of soil pH and driving factor analysis based on environmental variable screening. *Sustainability* **17**(7), 3173 (2025).

[78] Vijayan, K. Approaches for enhancing salt tolerance in mulberry (Morus L)—A review. *Plant Omics J.* **2**(1), 41–59 (2009).

[79] Jing, T. et al. Role of calcium nutrition in plant physiology: Advances in research and insights into acidic soil conditions—a comprehensive review. *Plant Physiol. Biochem.* **198**, 108602 (2024). 10.1016/j.plaphy.2024.108602 38608506

